# The clinical and public health problem of relapse despite primaquine therapy: case review of repeated relapses of *Plasmodium vivax* acquired in Papua New Guinea

**DOI:** 10.1186/1475-2875-13-488

**Published:** 2014-12-12

**Authors:** R Joan H Ingram, Chelzie Crenna-Darusallam, Saraswati Soebianto, Rintis Noviyanti, J Kevin Baird

**Affiliations:** Auckland City Hospital, Auckland, New Zealand; Eijkman Institute for Molecular Biology, Jakarta, Indonesia; Eijkman-Oxford Clinical Research Unit, Jakarta, 10430 Indonesia; Centre for Tropical Medicine, Nuffield Department of Medicine, University of Oxford, Oxford, UK

**Keywords:** *Plasmodium vivax*, Relapse, Primaquine, Resistance, Tolerance, Compliance, Therapeutic failure, CYP2D6 polymorphism

## Abstract

**Background:**

Primaquine is the only drug available for preventing relapse following a primary attack by *Plasmodium vivax* malaria. This drug imposes several important problems: daily dosing over two weeks; toxicity in patients with glucose-6-phosphate dehydrogenase (G6PD) deficiency; partner blood schizontocides possibly impacting primaquine safety and efficacy; cytochrome P-450 abnormalities impairing metabolism and therapeutic activity; and some strains of parasite may be tolerant or resistant to primaquine. There are many possible causes of repeated relapses in a patient treated with primaquine.

**Case description:**

A 56-year-old Caucasian woman from New Zealand traveled to New Ireland, Papua New Guinea for two months in 2012. One month after returning home she stopped daily doxycycline prophylaxis against malaria, and one week later she became acutely ill and hospitalized with a diagnosis of *Plasmodium vivax* malaria. Over the ensuing year she suffered four more attacks of vivax malaria at approximately two-months intervals despite consuming primaquine daily for 14 days after each of those attacks, except the last. Genotype of the patient’s cytochrome P-450 2D6 alleles (*5/*41) corresponded with an intermediate metabolizer phenotype of predicted low activity.

**Discussion:**

Multiple relapses in patients taking primaquine as prescribed present a serious clinical problem, and understanding the basis of repeated therapeutic failure is a challenging technical problem. This case highlights these issues in a single traveler, but these problems will also arise as endemic nations approach elimination of malaria transmission.

## Background

Endemic *Plasmodium vivax* puts 2.7 billion residents at risk of infection, and very many visitors [1]. In the USA, during 1990 to 2010, between 400 and 700 Americans were diagnosed with vivax malaria each year [2]. In the UK, between 2002 and 2005, a total of 1,244 cases of *P. vivax* malaria were reported [3]. Each primary attack carries risk of repeated attacks called relapses in the weeks and months to follow, for up to three years [4]. The probability, number, and timing of relapses vary by geographic origin of the strain [5]. Three to five relapses may be considered typical, and up to 20 within two years have been documented [6].

Despite long being considered relatively benign and very rarely fatal, recent work demonstrates the potential for serious morbidity and even mortality with vivax malaria [2, 7–11]. Severe anaemia is the most common threatening syndrome, followed by acute respiratory distress, kidney failure, severe thrombocytopaenia, coma, and shock syndromes. Severe and fatal complications in travellers also occur [7, 12]. Preventing repeated attacks of relapsing vivax malaria offers the opportunity to avoid threatening illness and onward transmission.

Primaquine alone has proven safety and efficacy against the latent parasite in the liver (hypnozoites) responsible for relapsing vivax malaria. Licensed in 1952, primaquine has been combined with chloroquine against the asexual blood stages to achieve radical cure, i.e., elimination of all forms of the parasite. A wide variety of primaquine regimens have been applied against relapse [13], but today experts agree that 0.5 mg/kg daily for 14 days should prevent relapse [14].

Primaquine toxicity in glucose-6-phosphate dehydrogenase (G6PD)-deficient patients imposes several important obstacles to achieving radical cure. Adherence to 14 days of daily dosing may be unlikely without firm guidance from the provider and follow up [15]. However, strict adherence invites risk of serious harm in most G6PD-deficient patients. So most providers do not offer primaquine therapy without knowing G6PD status [16]. When providers do offer primaquine, advice to fully adhere must include the caution to cease dosing with signs of haemolysis. A robust point-of-care diagnosis of G6PD deficiency would mitigate this problem of safe access to effective therapy against relapse, but not all of them.

Several other problems diminish effectiveness of radical cure by primaquine. Differential cytochrome P450 2D6 isozyme metabolism of primaquine by dozens of distinct allelic variants introduces highly complex determinants of clinical efficacy. Further, the rise of resistance to primaquine’s partner in radical cure, chloroquine, requires adopting other blood schizontocides as therapy, but the safety and efficacy of primaquine against relapse may vary when combined with those partner drugs in radical cure [17]. Each new partner therapy requires new evidence of primaquine safety and efficacy, and such is relatively difficult to obtain. Pregnant or lactating women, and infants may not receive primaquine therapy [18], and these groups are the most vulnerable to severe and threatening vivax malaria in endemic communities [19, 20].

Sixty years of continuous primaquine use requires consideration of the potential problem of resistance to it by hypnozoites. However, as the case described in this report illustrates, demonstrating either sensitivity or resistance to primaquine is a very complex, multi-tiered endeavour requiring the ruling out of many possible confounding factors. Repeated acute attacks of vivax malaria despite primaquine therapy poses threats to the health and life of the patient, and puts the care providers in the difficult position of identifying the cause of therapeutic failure in order to address it. These patients also raise public health issues, especially regarding strategies for eliminating malaria transmission.

Here, a summary of a case obtained from medical records and the first author’s clinical review of the patient is presented to highlight the issue of multiple relapses of *P. vivax* despite apparently adequate primaquine therapy.

## Case presentation

### Travel, attacks and treatment

A 56-year-old woman resident in New Zealand weighing 67 kg arrived at New Ireland, Papua New Guinea in early September 2012. During that visit she consumed 100 mg doxycycline daily up to the end of travel in late October 2012 and for one month following return to New Zealand. She reported being fully compliant with that chemoprophylaxis. She had previously experienced an episode of confirmed *Plasmodium falciparum* after travel to Africa and wished to avoid another such attack.

**Figure 1:**
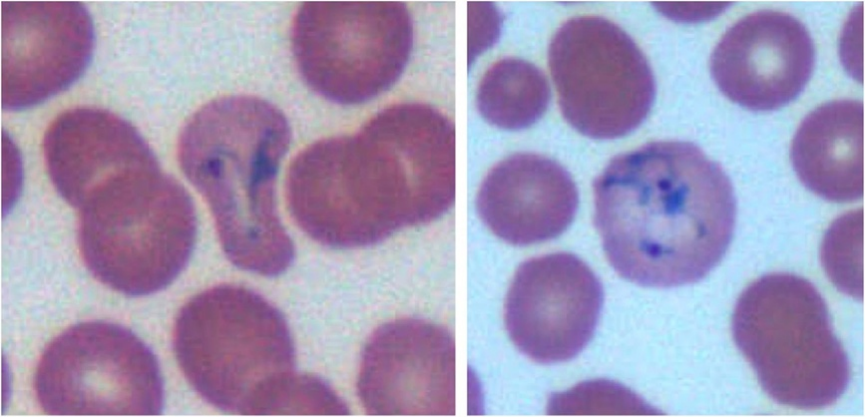
**Photomicrograph of an oil-immersion microscopic examination of a Giemsa-stained thin blood smear taken from the patient illustrating amoeboid trophozoites within enlarged and mutable infected red blood cells consistent with a diagnosis of infection by** ***Plasmodium vivax*** **.**

A week later, in November 2012, she sought medical attention after developing headache, malaise, and rigors. A diagnosis of *P. vivax* was made microscopically (Figure 1). She spent two days in hospital and was discharged after responding to therapy with atovaquone/proguanil (Malarone™, 4 tablets/day × 3 d). She was prescribed an unknown dose of daily primaquine (Primacin™, BNM®, Australia) for this first attack after her G6PD test was normal.

Two months later, in January 2013 she suffered a second attack, again confirmed with a microscopic diagnosis and with admission to hospital. She was treated with artemether/lumefantrine (Riamet™, 4 tablets twice daily for 3 days) and 30 mg primaquine base daily for 14 days.

Two months later, in March 2013, she suffered a third confirmed attack of *P. vivax* malaria and was hospitalized for several days. She received the same therapy as that prescribed after the second attack. The patient described being fully compliant to daily primaquine therapy with prior treatments. The importance of doing so was again emphasized to the patient.

A fourth attack occurred two months later in May 2013. She was admitted to hospital with a peripheral parasitaemia of asexual *P. vivax* trophozoites infecting 3-4% of her red blood cells. She was treated with the same regimen of therapy as after the second and third attacks, but with 45 mg primaquine daily for 14 days, rather than 30 mg. She spent one day in hospital.

The fifth attack again occurred two months later, in July 2013. She was not admitted to hospital and consumed artemether/lumefantrine as before. Primaquine therapy was judged unlikely to provide any benefit and was not prescribed.

In August 2013 the patient travelled to the Solomon Islands and remained for two months, all the while consuming a commercial dietary supplement containing *Artemisia annua* herbal extract (equivalent of 17 mg artemisinin daily) that had been recommended to her by a colleague for malaria prevention. She remained healthy while abroad. Five months after returning to New Zealand, she again suffered an attack of vivax malaria (March 2014). This was treated initially and partially with chloroquine (two doses) with a switch to atovaquone/proguanil, and primaquine was again not prescribed for the same reasons as in July 2013.

The patient travelled to Vanuatu for two weeks in April and May 2014, taking 100 mg doxycycline daily while there, and continues that regimen up to December 2014 with intervening travel to the Solomon Islands. As of December 2014 she has not suffered any attacks of malaria since March 2014, but has on three occasions developed severe headache, weakness and chills which have settled within 24 hours without medical attention.

Although these six episodes of vivax malaria were not complicated, each caused marked symptoms and most led to admission to hospital. These events were debilitating and disruptive of the patient’s routines, and she firmly declared that she took the four courses of primaquine precisely as directed.

### Plasmodium diagnostics

Giemsa-stained thin blood films were taken at each presentation with acute illness. Demonstration of amoeboid trophozoites within enlarged infected red blood cells provided the diagnosis of vivax malaria (Figure 1) at the laboratory. Expert malaria microscopists at the Eijkman-Oxford Clinical Research Unit in Jakarta, Indonesia affirmed that diagnosis. At the molecular diagnostic referral laboratory also in Jakarta (Eijkman Institute for Molecular Biology), a vial of extracted DNA from blood taken at the final attack of malaria was analyzed by nested PCR of ssrRNA using standardized methodology employing genus and species-specific primers (for *Plasmodium malariae, Plasmodium ovale, P. falciparum,* and *P. vivax*) [21]. These were strongly positive for *P. vivax* and negative for the other species.

### CYP2D6 genotyping

Isolated DNA was collected from the patient and extracted using Puregene™ (Gentra®) and its concentration measured at 635 ng/μL (NanoDrop2000™, Thermoscientific®) and 1.58 DNA purity (A260/A280). CYP2D6 genotyping was performed using xTAG™ CYP2D6 kit v3 (Luminex® Corporation, USA). Allelic variation categories assayed by this kit included DNA sequences encoding normal function (*1, *2, and *35), reduced function (*9, *10, *17, *29, and *41), and non-functional (*3, *4, *5 –for whole-gene deletion, *6, *7, *8, *11, and *15), including DUP for duplication. The manufacturer’s instructions were followed for genotyping by this kit. Amplification and incubation was performed using 9700 thermal cycler (PE Applied Biosystems®). The Luminex® 200 xMAP™ instrument was used to read the fluorescence absorbance from a specific probe that hybridized with the beads. The data were then analysed automatically using the xTAG Data Analysis Software™ to record the CYP2D6 allelic variant.

**Figure 2:**
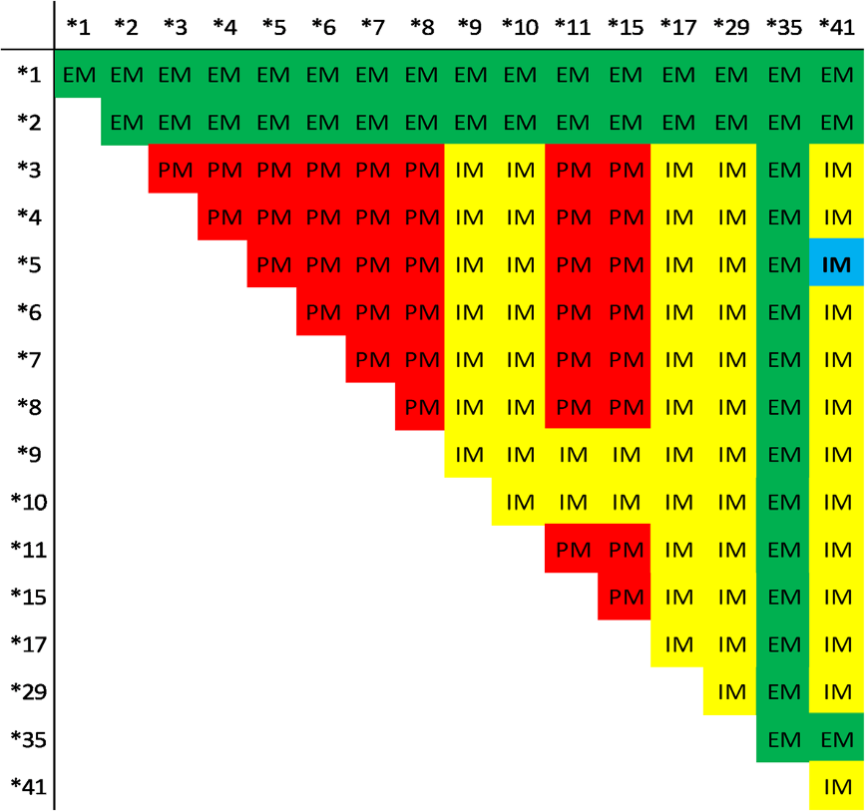
**Phenotype prediction from CYP2D6 allelic variants detected by the xTAG™ CYP2D6 Kit v3 (Luminex® Corp., USA).** Green corresponds to extensive metabolizer (EM), yellow to intermediate metabolizer (IM), and red to poor metabolizer phenotypes. Our patient’s genotype placement at *5/*41 is shown as blue in an intermediate metabolizer phenotype.

A CYP2D6 gene deletion (*5) and polymorphisms at 1661G > C, 2850C > T, 2988G > A, and 4180G > C (*41) were detected. The genotype of the patient was classified as a *5/*41 allelic variant. According to the prediction of enzymatic activity, the *5 and *41 allelic variants are categorized as null and dysfunctional, respectively [22, 23]. The combination of those allelic variants infers an intermediate metabolizer phenotype (Figure 2). Nonetheless, *41 allele is frequently associated with relatively very low enzymatic activity scores when expressed with the null *5, i.e., 0.5 on a scale of 0 to 3, where 3 is the ultra-metabolizer, and 1.5 to 2.0 represent wild type *1/*1 scores [24]. Absent direct characterization of CYP2D6 metabolizer phenotype – as may be done with a dextromethorphan challenge and pharmacokinetic analysis [25] – it can only be considered it likely that the CYP2D6 genotype in this patient resulted in levels of metabolized primaquine inadequate to the task of killing hypnozoites of *P. vivax*. Likewise, one research subject experimentally challenged with *P. vivax* and relapsing despite directly observed high-dose primaquine therapy had *4/*41 genotype of CYP2D6 [26], where *4 has no functional enzyme activity (like the *5 deletion) [23].

### Interpretation

This patient suffered an attack of *Plasmodium vivax* post-travel, followed by four relapses, each at two-month intervals, and a fifth attack more than six months after the prior attack and intervening travel to another malaria endemic area. She consumed full courses of primaquine after the first, second, third, and fourth attacks, including a round of 45 mg/day for 14 days. None of these prevented her from relapsing at intervals typical of *P. vivax* from that region [5]. The challenge for her care providers, and malariologists weighing strategies for elimination of this parasite, is to understand what failed in this case and what may be done to avoid such poor therapeutic outcomes in the future.

The *Plasmodium vivax* strain called Chesson came from an American soldier infected in 1944 during the New Guinea campaign of the Pacific War [27]. This strain and most from the Pacific region relapse quickly (usually within a month of onset of the primary attack) and at approximately two-month intervals [28]. Chesson *P. vivax* also tolerated 14-day courses of primaquine at 0.25 mg/kg daily, necessitating 0.5 mg/kg daily for good efficacy [29]. The parasite infecting the patient described here also came from the New Guinea region and appears Chesson-like in its relapsing behaviour, and proved unresponsive to even 0.75 mg/kg daily primaquine therapy for 14 days. Understanding why primaquine failed in this patient requires considering many possible explanations, including failure to take therapy as directed, inappropriate partner blood schizontocide, counterfeit or substandard primaquine, re-infection, or polymorphisms in the sole human cytochrome P-450 isozyme (2D6, or CYP2D6) [30] known to metabolize primaquine [31], an essential step in killing hypnozoites [32].

Bennett and colleagues [26] challenged 25 human subjects with *P. vivax* sporozoites, and all suffered a primary attack and were treated with directly observed standard chloroquine and primaquine (0.5 mg/kg/d × 14 d) radical cure. Two subjects nonetheless relapsed. The CYP2D6 genotypes of those two subjects were classified as associated with intermediate or poor metabolizers, respectively. The intermediate metabolizer relapsed twice, and the poor metabolizer relapsed three times. Among the 23 subjects who did not relapse, only two were classified as intermediate metabolizers, and the remaining 21 subjects were extensive metabolizers. Higher levels of un-metabolized primaquine in plasma among the defective metabolizers suggested far lower rates of conversion to active metabolites. These findings tended to rule out parasite resistance to primaquine as the explanation of the therapeutic failures. Taken with the facts of directly observed therapy and no possible re-infection, those studies pointed to CYP2D6 polymorphisms as being important determinants of efficacy against relapse.

Likewise, a specific CYP2D6 genotype in this patient (*5/*41) seems to account for the poor outcomes of her repeated rounds of primaquine therapy. The parasite infecting the patient relapsed at its naturally short intervals (two months), seemingly unaffected by the primaquine being consumed. Primaquine exerts a cumulative dose effect on hypnozoites called the total dose effect, where the total dose determines efficacy rather than the schedule of delivery [33]. In other words, the same total dose may be delivered as single dose, as 7 or 14 daily doses, or as weekly doses for eight weeks and achieve the same good efficacy [34]. Whereas a total dose of 420 mg is considered efficacious against even the primaquine-tolerant Chesson strain of *P. vivax*, this patient received a total dose of 1,890 mg over an eight-month period, relapsing repeatedly in that interval. Even this very large cumulative total dose appeared inadequate against relapse.

Another possible explanation for therapeutic failure is the role of unproven partner blood schizontocides given to this patient. Her care providers prescribed atovaquone-proguanil and, later, artemether-lumefantrine. Resistance to chloroquine by *P. vivax* in the Asia-Pacific region is widely known [35]. The safety and efficacy of primaquine against relapse depends on the partner blood schizontocide [17]. No clinical trials have proven primaquine safe and effective when used with either of those blood schizontocidal therapies as partner in radical cure. Drug-drug interactions between those therapies somehow disabling therapeutic efficacy against relapse cannot be ruled out in this patient. Poor adherence or faulty primaquine cannot be ruled out, but are considered very unlikely. The patient’s CYP2D6 polymorphism was considered the most likely basis of her experiences with *P. vivax*.

### Implications

Primaquine has been the sole therapy against relapse of vivax malaria since 1952. The parasite has certainly been exposed to sub-therapeutic levels over that period [13]. Acquired resistance to primaquine must be regarded as a possibility, even though hypnozoite resistance to primaquine has yet to be demonstrated in animal models, patients, or study subjects. This may be a product of resistance being absent, or of the difficulty of ruling out all other possible causes of therapeutic failure.

No standardized in vivo protocol has been developed, optimized or validated for the diagnosis of resistance to primaquine in a patient. Re-infection would seriously confound the assessment of primaquine sensitivity, and no molecular diagnostics today differentiate relapse from reinfection. Randomized clinical trials can measure the force of infection of blood via hypnozoites versus sporozoites in populations [36], but such trials are extremely laborious and expensive, and not practical for routine surveillance. All of these difficulties explain why we cannot now conduct surveillance for primaquine resistance/efficacy in endemic areas. Thus, primaquine efficacy in most settings cannot be known. An analysis of 87 primaquine therapeutic trials since 1950 by John *et al.*[37] illustrates an attempt at doing so despite the limitations and confounders involved.

Only under relatively rare circumstances – as in experimental challenge or in mobile populations from non-endemic areas being briefly heavily exposed and then repatriated – can the efficacy of primaquine be measured with little or no ambiguity. In one such instance, the efficacy of primaquine against hypnozoites acquired in Indonesian New Guinea was a remarkable 98% at 0.5 mg/kg/day X 14d following dihydroartemisinin-piperaquine as partner blood schizontocide [38]. That one study offers hope of good efficacy elsewhere, but does not substitute for a responsibly thorough and effective means of surveillance for resistance to primaquine.

Travellers returning home and suffering vivax malaria may be the sole means now available to reliably estimate the efficacy of primaquine against relapse in any given area. The technical difficulty of interpreting outcomes in this patient in New Zealand illustrates the complexity in understanding the broader determinants of therapeutic efficacy against relapse. The extraordinary diversity and complexity of CYP2D6 polymorphisms will require a great deal more attention in the context of malaria control and elimination. The current case illustrates the value of CYP2D6 assessment in patients needing primaquine therapy.

## Conclusions

The findings in this case review are compatible with therapeutic failure of primaquine as a consequence of a polymorphism of CYP2D6 (*5/*41) impairing metabolism to an active form. Unproven combinations of ACT therapies with primaquine for radical cure exacerbating therapeutic inadequacy may not be ruled out, along with other factors like adherence and sub-standard drug. Defining the determinants of primaquine therapeutic failure against relapse clarifies strategy aimed at achieving maximal effectiveness of primaquine against the hypnozoite reservoir of *P. vivax*. Elimination of vivax malaria may hinge upon success at doing so.

## Consent

Written informed consent was obtained from the patient for publication of this case study and accompanying images. A copy of the written consent is available for review by the Editor-in-Chief of this journal.

## Abbreviations

ACT: Artemisinin combined therapy
ASPE: Allele-specific primer extension
CYP: Cytochrome P-450
G6PD: Glucose-6-phosphate dehydrogenase.
